# The Effects of Soy Bean Flour Enriched Bread Intake on Anthropometric Indices and Blood Pressure in Type 2 Diabetic Women: A Crossover Randomized Controlled Clinical Trial

**DOI:** 10.1155/2014/240760

**Published:** 2014-09-08

**Authors:** Asma Salari Moghaddam, Mohammad Hassan Entezari, Bijan Iraj, Gholamreza Askari, Elham Sharifi Zahabi, Mohammad Reza Maracy

**Affiliations:** ^1^Food Security Research Center, Isfahan University of Medical Sciences, P.O. Box 81745-151, Isfahan, Iran; ^2^Department of Clinical Nutrition, School of Nutrition and Food Science, Isfahan University of Medical Sciences, P.O. Box 81745-151, Isfahan, Iran; ^3^Endocrine and Metabolism Research Center, Isfahan University of Medical Sciences, P.O. Box 81745-151, Isfahan, Iran; ^4^Department of Community Nutrition, School of Nutrition and Food Science, Isfahan University of Medical Sciences, P.O. Box 81745-151, Isfahan, Iran; ^5^Department of Epidemiology & Biostatics, School of Public Health, Isfahan University of Medical Sciences, P.O. Box 81745-151, Isfahan, Iran

## Abstract

Previous studies showed that soy bean has the potential to improve many aspects of diabetes state and provide metabolic benefits that aid in weight management. We aimed to determine the effects of soy bean flour enriched bread on anthropometric indices and blood pressure among type 2 diabetic patients. This randomized, crossover, clinical trial was performed in 30 type 2 diabetic women. There were two trial periods for 6 weeks and a wash-out period for 4 weeks. In the soy bread diet period, 120 g of soy bean flour enriched bread was consumed each day instead of the same amount of their usual bread or other cereal products. After a 4-week wash-out period, participants were crossed over for another 6 weeks. Mean (±SD) age of study participants was 45.7 ± 3.8 years. The results of our study showed no significant effects of soy bean flour enriched bread on anthropometric indices and blood pressure among diabetic patients. Despite the slight reduction in BMI, waist circumference, and percent of body fat, there were no significant differences in changes of these values between two groups. No significant changes in waist to hip ratio and blood pressure were seen.

## 1. Introduction

Diabetes mellitus is one of the most common chronic diseases in the world [1] and has become a major threat for global health [2]. It has been estimated that the prevalence of diabetes for all age-groups worldwide was 2.8% in 2000 and will reach 4.4% in 2030 [3]. According to the World Health Organization (WHO) estimates, more than 2 million diabetic patients were living in Iran in 2000 and has been estimated to increase to more than 6.4 million in 2030 [4]. Currently, the effects of soy and its components on many chronic diseases have been studied. Soy bean is a legume which is a unique source of protein, fiber, vitamins, minerals, polyunsaturated fatty acids, isoflavones, and phytoestrogens [5, 6]. The effects of soy on body composition are not well understood. In a cross-sectional study conducted in postmenopausal women, individuals who received a high soy diet had a lower BMI and waist circumference compared with individuals who received no soy [7]. In contrast, several studies have failed to reach such significant effects. In a randomized trial in perimenopausal women, soy protein did not affect total body fat or lean mass [8]. The same results were found in a 6-week crossover study in overweight and obese female youths, which soy drink replacement had no significant effects on weight and waist circumference [9]. Soy contains high fiber content. Earlier studies have shown that higher intake of dietary fiber is linked to suppressed appetite and enhanced satiety [6]. Besides, soy is a rich source of protein (~35–40%) [10]. Recently, protein intake is considered as a major determinant in weight control [11]. High protein content of foods can reduce appetite and decrease food intake [12]. Also soy protein can reduce appetite by stimulating cholecystokinin [13]. Soy polyphenols can affect endothelial function and as a consequence, blood pressure [14]. Prevalence of type 2 diabetes is increasing in Iran [4]. It seems that the inclusion of soy foods in the diet is particularly relevant for diabetic patients because soy foods have beneficial effects on chronic diseases and soy can help them in weight management and then can improve many aspects of diabetes state [5, 15]. In addition, bread is the staple food in Iranian diet. Fortification of bread with soy flour can increase the quality of protein and improve its effects on human health. According to our knowledge, there is no study examining the effects of soy bean flour enriched bread on weight and blood pressure control among women with type 2 diabetes. Therefore, With regard to beneficial effects of soy on chronic disease including type 2 diabetes, we aimed to fortify bread with soy bean flour and determine its effects on anthropometric indices and blood pressure among type 2 diabetic patients.

## 2. Subjects and Methods

### 2.1. Participants

**Figure 1:**
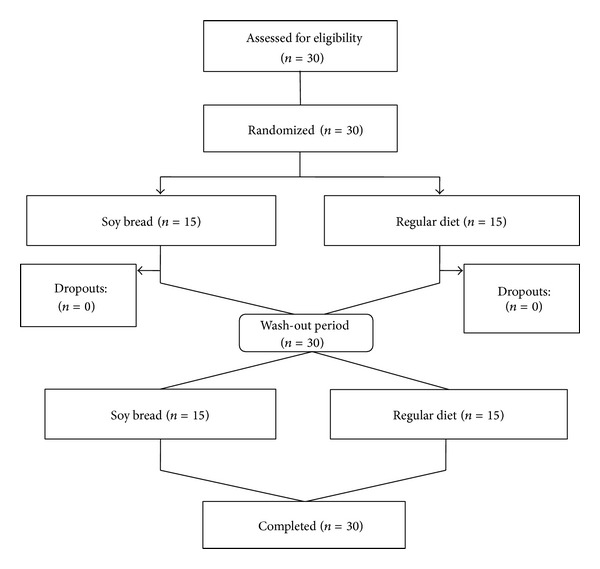
Participants flow diagram.

This randomized, crossover, controlled clinical trial was undertaken in 30 premenopausal women with type 2 diabetes. This study was conducted in Isfahan, Iran, from April 2013 to September 2013. Being in the range age of 30–50, diagnosis of type 2 diabetes and having body mass index >25 were the inclusion criteria. Exclusion criteria were insulin injection, use of hormone replacement therapy, use of supplements, hypo- and hyperthyroidism, smoking, and allergy to soy bean. Participants who were pregnant or breastfeeding were excluded. All participants were recruited from Endocrine and Metabolism Research Center of Isfahan University of Medical Sciences. Participants were not undergoing dietary changes in the last 3 months and had no current weight loss. Sample size for this study was calculated based on suggested formula for crossover trials [16]: *n* = [(*z*_1−*α*/2_+*z*_1−*β*_)^2^ · *s* ^2^]/2Δ^2^; we considered type 1 error of 5% and type 2 error of 20% (power = 80%) and BMI as a key variable [9]. According to the previous formula, 19 participants were needed for adequate power. Since there are high dropouts in crossover trials, we enrolled 30 women in this study based on the above mentioned inclusion criteria. All participants completed the entire crossover study. Participants diagram is shown in Figure 1. This study was approved by Ethical Committee of Isfahan University of Medical Sciences, Isfahan, Iran. All participants provided informed written consent. The clinical trial registration code was obtained from the center for registration of clinical trial (IRCT2013061613684N1).

### 2.2. Study Procedures

**Figure 2:**
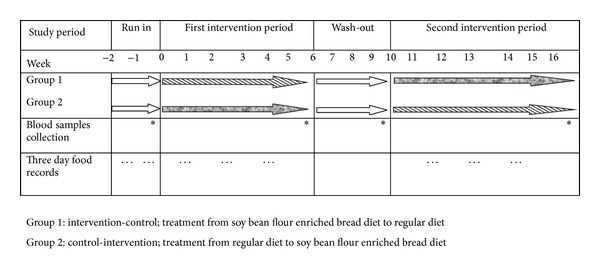
Study diagram.

At first, a 2-week run-in period was conducted. This period was conducted to assess diet and physical activity level and to evaluate the compliance of study participants to soy bean flour enriched bread. Participants were asked not to alter their habitual diet and level of physical activity. Also, to improve compliance, participants were asked to consume 1 serving/day of soy bean flour enriched bread during this period of the study. In the run-in period, all participants completed two dietary records (nonconsecutive days) and a 2-day physical activity record. After run-in period, all measurements were done. Then, participants were randomly assigned to either intervention or control groups, each one for 6 weeks. Participants were not blinded because of texture and taste of the bread. After the first period of intervention, a 4-week wash-out period was conducted. Then participants were crossed over for another 6 weeks. During the wash-out period, subjects consumed the same diet they consumed before the study. All measurements were done at baseline and at 6, 10, and 16 weeks. Compliance of the participants monitored once a week through face-to-face visits and phone interviews. In addition, all participants completed a 3-day (2 weekdays and one weekend day) dietary and physical activity records once every two weeks during the study. Study diagram is shown in Figure 2.

### 2.3. Interventions

Soy bean flour enriched bread was prepared by replacing 30% of the wheat flour by soy bean flour. Participants in the intervention group (soy bread) were asked to consume 120 g of soy bean flour enriched bread each day instead of the same amount of their usual bread intake and if necessary other carbohydrate rich foods such as rice, pasta and other cereal products. On average, each bread was 120 g. Participants were supplied with enough fresh packaged bread weekly. Bread packages used fresh or were frozen before use. Individuals in the control group were asked to remain on their habitual diet. The dietitian monitored bread intake weekly, if bread intake was outside the recommended amount. Participants were trained on how to use bread properly. Soy bean flour enriched bread characteristics are shown in Table 1.

### 2.4. Anthropometric Assessments

Height was measured to the nearest 0.1 cm in a standing position without wearing shoes, using a measuring tape while shoulders were relaxed. Weight was measured to the nearest 0.1 kg using a digital scale with minimal clothes and without shoes (Seca, Hamburg, Germany). Body mass index (BMI) was calculated as weight (in kg) divided by height^2^ (in m). Waist circumference (WC) was measured to the nearest 0.1 cm at the narrowest level over light clothing, by using a nonstretchable tape measure, without any pressure to the body surface. Hip circumference (HC) was measured in the largest part of the hip over light clothing. Percent of body fat (PBF) was measured by body composition analyzer (Jawon Medical Company, Korea). Blood pressure was measured after 15 minutes of rest in the seated position by the use of a mercurial sphygmomanometer.

### 2.5. Statistical Methods

Dietary records were assessed using Nutritionist IV software. Statistical analyses were done using SPSS 18 statistical software package (SPSS Inc., Chicago, IL). All subjects were included in the final analysis. At first, normal distribution of all variables was checked with the QQ-Plot test. Paired *t*-test was used to examine the main effects by comparing the mean differences of variables in two groups. Carry over effect and period effect were checked using *t*-test. All means are presented as mean ± standard deviation ($\overset{-}{x}\pm \text{SD}$). Values of *P* < 0.05 were considered as statistically significant.

## 3. Results

Mean (±SD) age of study participants was 45.7 ± 3.8 years. Mean BMI and waist circumference was 29.5 ± 3.9 kg/m^2^ and 87.4 ± 6.7 cm, respectively. Dietary intakes of participants throughout the study showed that participants in the intervention group received higher energy compared with the control group, but the difference was not significant. No significant differences in dietary intakes of macronutrients, SFA, PUFA, MUFA, calcium, magnesium, folate, and vitamin C were observed between the two groups.

No adverse effects from soy bean flour enriched bread consumption were reported during the study. The effects of soy bean flour enriched bread intake on anthropometric indices and blood pressure are presented in Table 2. The results of our study showed no significant effects of soy bean flour enriched bread on anthropometric indices and blood pressure. Despite the slight reduction in BMI (change difference: −0.05, *P* = 0.8), waist circumference (change difference: −0.55, *P* = 0.26), and percent of body fat (change difference: −0.36, *P* = 0.45), there were no significant differences in changes of these values between two groups. No significant changes in waist to hip ratio and blood pressure were seen.

## 4. Discussion

The results of the present study conducted among type 2 diabetic women showed that daily consumption of 120 g soy bean flour enriched bread for 6 weeks could not significantly affect anthropometric indices and blood pressure. To our knowledge, this is the first interventional study that examined the effects of soy bean flour enriched bread intake on anthropometric indices and blood pressure among diabetic women.

Obesity is a growing concern due to its increasing prevalence and obesity related health problems [17]. Recently, nutritional strategies for weight management have attracted a great deal of attention. Due to high protein and low cholesterol content, soy is often used as a dietary component in weight loss diets [18–20]. Most of diabetic patients are overweight or obese. Studies showed that dry beans intake such as soy bean has the potential effect to improve many aspects of diabetes state and provide metabolic benefits that aid in weight management. In the current study, we found that soy bean flour enriched bread intake for 6 weeks could decrease BMI, WC, and percent of body fat but these changes were not significant. In line with our study, findings of Kok et al.'s study do not support the hypothesis that soy isoflavones have favorable effects on body composition [21]. In contrast to our study, findings from cross-sectional studies have demonstrated an inverse association between soy genistein intake and BMI, waist circumference, and total body fat among postmenopausal women [7, 22]. Such findings have also been reported by another interventional study [23]. Several studies support the hypothesis that soy protein or soy phytoestrogens may be beneficial in prevention of obesity and diabetes [24, 25]. In one study conducted in overweight and obese subjects, meal replacement diet with high soy protein drink was effective in reducing weight and improving anthropometric indices [26]. In line with our study, there are some studies that showed no significant effects of soy intake on anthropometric measures and fat mass loss [27, 28]. Several mechanisms have been proposed to underlie the beneficial effects of soy intake on body weight and satiety regulation. Soy peptides may play a role on body weight control, possibly by increasing energy utilization [29]. Also soy phytoestrogens might have beneficial effects on reducing fat accumulation [30]. Soy may exert its favorable effects through its high fiber content. Dietary fiber intake stimulates gastrointestinal hormones secretion that may act as satiety factors [31]. Furthermore, soy proteins can manage appetite. Proteins suppress food intake and contribute to satiety and delay return of hunger compared with fat and carbohydrate. Mechanisms of protein that act on food intake include slowing gastric emptying and direct or indirect stimulation of gastrointestinal hormones such as cholecystokinin and glucagon like peptide-1 [13].

In the present study, we found that soy bean flour enriched bread for 6 weeks could not substantially affect blood pressure among diabetic women. This finding is in contrast to an earlier study in overweight and obese women, where soy drink consumption could favorably influence blood pressure [9]. In a cross-over clinical trial by Miraghajani et al., soy milk consumption for 4 weeks in 29 type 2 diabetic patients with nephropathy could decrease blood pressure [32]. Soy polyphenols can affect endothelial function and then blood pressure [14]. Evidence suggests a small beneficial effect of protein on blood pressure, especially for plant protein [33]. Soy protein intake increases nitric oxide levels that have vasodilatory effects. High amount of arginine, nitric oxide precursor, in the amino acid profile of soy protein might explain soy protein effects on nitric oxide levels [34]. Angiotensin-converting enzyme inhibitory peptides exist in plant proteins such that soy can be enzymatically released from precursor proteins. These peptides can reduce blood pressure by decreasing the vasoconstrictory effects of angiotensin II and enhancing the vasodilatory effects of bradykinin [35]. Our study has strengths as well as limitations. The main strength point of our study is the cross-over design of the study. Other strength points are high percentage of participants who completed the study and took 3-day food records throughout the study to assess compliance of participants to soy bean flour enriched bread. Furthermore, among different soy products, we used soy bean flour enriched bread, a novel food ingredient, for this intervention. Despite the strength points, there are some limitations which deserve attention. Soy effects may be associated with the duration of intervention. Short duration of our intervention might result in the lack of observing any significant effect of soy bean flour enriched bread on anthropometric indices and blood pressure. Further studies with longer duration might be needed.

It was not possible for us to design a double-blind study due to texture and taste of the bread. Also, daily distribution of bread package was impossible. The effects of soy on anthropometric indices and blood pressure may be linked to the dosage of soy. We were not capable of increasing the soy bean flour dosage in bread more than 30% because of unfavorable effects on texture and taste of the bread. In addition, as the trial performed among only women, we cannot generalize the results to the general population.

## 5. Conclusion

In conclusion, our findings suggest that daily intake of 120 g soy bean flour enriched bread for 6 weeks had no significant effects on anthropometric indices and blood pressure among type 2 diabetic women. Short duration of our intervention might result in the lack of observing any significant effect. Further studies with longer duration are warranted.

## Figures and Tables

**Table 1:** Characteristics of soy bean flour enriched bread used in the intervention.

Nutrient	Amount per 100 g
Fat	7.2
Carbohydrate	44.31
Protein	14.1
Moisture	28.24
Ash	2.5

**Table 2:** The effects of soy bean flour enriched bread intake on anthropometric indices and blood pressure in type 2 diabetic women^1^.

Variables	First period		Second period		Change differences	*P* ^4^
	Baseline	6th week	10th week	16th week		
Weight (kg): intervention-control^2^	71.1 ± 10	71.1 ± 9.7	70.8 ± 9.5	70.5 ± 10.1	0.12	0.7
Weight (kg): control-intervention^3^	76 ± 11.2	75.7 ± 9.5	75.6 ± 9.9	76 ± 9.6		
BMI (kg/m^2^): intervention-control	28.6 ± 3.6	28.6 ± 3.5	28.5 ± 3.3	28.1 ± 3.8	−0.05	0.8
BMI (kg/m^2^): control-intervention	30.2 ± 4.3	30.1 ± 3.7	30 ± 3.8	30.2 ± 3.8		
WC (Cm): intervention-control	87 ± 6.7	86.6 ± 6.3	86.5 ± 6.3	86.7 ± 6.3	−0.55	0.26
WC (Cm): control-intervention	89.4 ± 7.7	88.8 ± 7.2	88.6 ± 6.9	89.1 ± 6.5		
HC (Cm): intervention-control	98.5 ± 4.6	98.6 ± 4.6	98.6 ± 4.5	99.08 ± 4	−0.4	0.25
HC (Cm): control-intervention	99.3 ± 4.8	99.1 ± 4.7	99.8 ± 5.5	99.2 ± 4.3		
WHR: intervention-control	0.88 ± 0.04	0.88 ± 0.04	0.88 ± 0.03	0.88 ± 0.04	0.006	0.08
WHR: control-intervention	0.88 ± 0.04	0.88 ± 0.04	0.88 ± 0.04	0.88 ± 0.04		
PBF: intervention-control	36.8 ± 3.8	36.4 ± 3.6	36.5 ± 3.6	36.6 ± 3.5	−0.36	0.45
PBF: control-intervention	37 ± 4	36.6 ± 4.1	36.5 ± 3.9	36.9 ± 3.6		
DBP: intervention-control	76.7 ± 10.5	74.7 ± 8.3	73.3 ± 6.2	73.3 ± 4.9	3	0.1
DBP: control-intervention	72.7 ± 5.9	70 ± 6.5	73.3 ± 9.7	74.7 ± 7.4		
SBP: intervention-control	114.7 ± 11.2	114.7 ± 9.1	114.7 ± 6.4	114 ± 7.4	2	0.3
SBP: control-intervention	112.7 ± 7	112 ± 6.8	110 ± 8.4	114 ± 9.8		

^1^All data are means ± SD.

^2^Intervention-control; treatment from soy bean flour enriched bread diet to regular diet.

^3^Control-intervention; treatment from regular diet to soy bean flour enriched bread diet.

^4^Results of paired *t*-test (mean differences between groups).

BMI, body mass index; WC, waist circumference; WHR, waist to hip ratio; PBF, percent of body fat; DBP, diastolic blood pressure; SBP, systolic blood pressure.
